# A Music Emotion Classification Model Based on the Improved Convolutional Neural Network

**DOI:** 10.1155/2022/6749622

**Published:** 2022-02-14

**Authors:** Xiaosong Jia

**Affiliations:** ^1^College of Music and Dance, JiNing Normal Unisersity, JiNing, Inner Mongolia 012000, China; ^2^Philippine Christian University, Manila, Philippines

## Abstract

Aiming at the problems of music emotion classification, a music emotion recognition method based on the convolutional neural network is proposed. First, the mel-frequency cepstral coefficient (MFCC) and residual phase (RP) are weighted and combined to extract the audio low-level features of music, so as to improve the efficiency of data mining. Then, the spectrogram is input into the convolutional recurrent neural network (CRNN) to extract the time-domain features, frequency-domain features, and sequence features of audio. At the same time, the low-level features of audio are input into the bidirectional long short-term memory (Bi-LSTM) network to further obtain the sequence information of audio features. Finally, the two parts of features are fused and input into the softmax classification function with the center loss function to achieve the recognition of four music emotions. The experimental results based on the emotion music dataset show that the recognition accuracy of the proposed method is 92.06%, and the value of the loss function is about 0.98, both of which are better than other methods. The proposed method provides a new feasible idea for the development of music emotion recognition.

## 1. Introduction

With the rapid development of the computer network and multimedia technology, more and more multimedia data such as text, image, audio, and video emerge in the internet. The management and analysis of media data have also become a hot issue [1]. As an important part of multimedia data, music works show explosive growth in both quantity and type. Music emotion recognition (MER) has important research value in the fields of music database management, music retrieval, music recommendation, and music therapy, and it has attracted extensive attention of experts and scholars [2, 3]. As an important emotional carrier, music contains rich emotional semantic information. Emotional words are the most commonly used words in retrieving and describing music. Therefore, classifying music with emotion as a label can effectively improve the efficiency of music retrieval and has gradually become a research hotspot [4].

At present, the research on music emotion recognition is mainly divided into two aspects. The first is how to better extract the emotional features of music. The accurate extraction of music emotional features is directly related to the accuracy and efficiency of classification results [5, 6]. The second is how to improve the classifier performance of emotion recognition. Because MER involves both music psychology and computer science, it is difficult to realize music emotion classification [7].

At present, the purely manual way of marking music emotion is inefficient, and the quality cannot be guaranteed, which is difficult to meet the emotional labeling needs of a large number of music works [8]. Therefore, more and more experts begin to study the automatic music emotion recognition technology [9]. Nordström and Laukka [10] conducted in-depth research on emotion recognition. The research results show that emotion recognition points reflect the amount of information required for stable recognition. The anger and happiness emotion recognition points of voice and music are the shortest, but the recognition of music and speech takes longer to be stable. At the same time, there is a positive correlation between voice and music, indicating that there is a common sound code to express emotion. Most of the traditional music emotion classification methods focus on the analysis of lyrics or audio. For example, Bo et al. [11] studied the emotional cognitive process and its application. The time course analysis method of music-induced emotion is proposed to investigate the difference of brain activity. Based on the EEG characteristics inspired by cognitive principles, a music emotion classification system was constructed. However, the single-mode data can only get part of the object's characteristics. There was a certain degree of information loss by classifying data only using single-mode data [12]. In recent years, more and more researchers have begun to pay attention to multimodal fusion technology, which also provides a new solution for music emotion classification [13]. Zhang et al. [14] explored a new method of personal music emotion recognition based on human physiological characteristics. A feature database based on music-related emotions and a database based on physiological signals generated by listening music were established. The results showed that the proposed method has good performance. However, the robustness and emotional sensitivity to genre, timbre, and noise changes are not strong.

Intelligent classification method based on machine learning is gradually applied to music emotion classification [15]. Hizlisoy et al. [16] proposed a music emotion recognition method based on the convolutional long- and short-term memory depth neural network. The performance of this method is evaluated on the constructed database. The logarithmic mel-filter banks and mel-frequency cepstral coefficient were used to provide features to the convolutional neural network layer to realize feature classification. The classification results in the constructed emotional music database showed that this method has good performance. However, the classification training of datasets still needs to occupy large computing resources and classification time. In [17], through the linear regression model, the acoustic features of music were mapped to the corresponding arousal and titer emotional indicators to realize the recognition task. However, the robustness and generalization ability of audio feature extraction are poor. Dong et al. [18] proposed a new bidirectional convolutional recursive sparse network for music emotion recognition based on the convolutional neural network and recursive neural network. The model adaptively learned the emotional salient features containing sequence information from the spectrum of the audio signal and realized the continuous emotional prediction of audio files by combining feature extraction and adaptive selection, but the performance of music in nonlinear deep semantic features needs to be improved. Rajesh and Nalini [19] proposed a new method to identify emotions through instrument categories using deep learning technology. The music datasets of four types of musical instruments were collected. Based on the extracted features, the recurrent neural network (RNN) was trained to identify emotions. The results showed that mel-frequency cepstral coefficient (MFCC) with the deep RNN achieved better performance for instrument emotion recognition, but it is difficult to be widely used in large-scale tasks due to higher complexity.

Based on the above analysis, aiming at the problem that single-mode data cannot fully express music emotion, a music emotion recognition method using the convolutional neural network is proposed. Compared with the traditional emotion classification methods, the innovation of the proposed method is as follows:

1. In order to obtain more comprehensive music features, the proposed method uses the convolutional recurrent neural network (CRNN) and bidirectional long short-term memory (Bi-LSTM) network to extract audio sequence features and context information, respectively, and fuse them for emotion classification to improve the accuracy of emotion recognition
2. The improved loss function combines the center loss and the distance between classes, which can improve the distinguishability of features, ensure the reduction of the distance within feature classes and increase the discrimination between different classes, and enhance the adaptability of fine-grained image classification

## 2. Algorithm Model

### 2.1. Overall Framework

**Figure 1:**
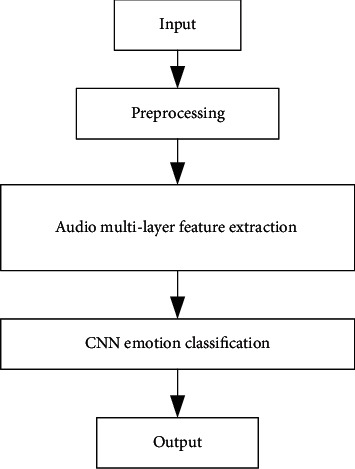
Overall framework of the proposed method.

Generally speaking, after extracting audio low-level features from audio signals, machine learning method is used for music emotion classification. At present, the performance of the music emotion recognition system based on audio meets the “ceiling,” which is because the low-level features of audio lack emotional relevance, and the high-level features are closer to emotion, but the manual design cost is high. Applying the deep learning method to audio emotion classification helps to bridge the semantic gap between audio low-level features and music high-level emotion concepts [20]. CRNN is suitable for sequence data modeling. Applying the CRNN to music emotion classification, a music emotion classification model based on the improved CNN is proposed. The overall architecture of the model is shown in Figure 1. Compared with the low-level features of audio, the spectrogram contains more audio information. Therefore, the model combines the spectrogram and the low-level features of audio as the input sequence to realize information complementarity.

The input of the improved CNN is sound spectrogram, in which the CNN uses two different convolution kernels to extract the time-domain and frequency-domain features of audio, respectively, and Bi-LSTM further extracts the sequence features of audio. The input of Bi-LSTM is audio low-level descriptors (LLDs) to obtain the sequence information of audio features. Finally, the feature connection of the CRNN and Bi-LSTM is used for emotion classification.

### 2.2. Audio Low-Level Feature Extraction

#### 2.2.1. Mel-Frequency Cepstral Coefficient (MFCC)

At present, content-based acoustic features are mainly divided into timbre, rhythm, pitch, harmony, and time features. Timbre features include cepstrum features, such as MFCC; rhythm features mainly include beat number and rhythm histogram; pitch features are mainly frequency information; harmony features include chromaticity diagram; time characteristics include time centroid. MFCC makes use of the auditory principle and the decorrelation characteristics of the cepstrum.

In order to extract MFCC features, first, the audio signal is preprocessed and windowed. The Blackman–Harris window is used to divide the original signal with a sampling rate of 44.1 kHz into 2048 sample frames. After windowing the audio signal, the two ends of each frame signal will gradually become 0, so the two ends of the signal will be weakened [21]. In order to overcome this problem, adjacent frames can overlap during frame division. Generally, the overlap length is half of the frame length or fixed as 10 ms. In the proposed method, the adjacent frames overlap by 50%, which can not only reduce the spectrum leakage but also reduce the unnecessary workload. Then, the spectrum energy is obtained by discrete short-time Fourier transform on each frame, next weighted by *κ*_1_ mel-filter frequency response, and further filtered to generate the mel spectrum. Its center frequency and bandwidth roughly match the auditory critical band filter. Finally, the whole mel spectrum sequence is divided into *L* blocks with the size of *κ*_2_ frames, which are represented as *Z*_*l*_,  *l*=1, *L*, *L* along the time axis. Therefore, the size of each block is *κ*_1_ × *κ*_2_.

#### 2.2.2. Residual Phase (RP)

Residual phase (RP) is defined as the cosine of the phase function of the analytical signal derived from the linear predictive (LP) residual of the music signal. At time *t*, the music sample *h*(*t*) can be estimated as a linear combination of the past *p* samples, so the predicted music sample can be expressed as

(1) $$\begin{array}{c} \dot{h}\left(t\right)=\sum_{i=1}^{p}a_{i}h\left(t-i\right), \end{array}$$

where *p* is the predicted time sequence and coefficient {*a*_*i*_}, *i*=1,2, *L*, *p*, is the set of linear prediction coefficients (LPCs).

The prediction error *e*(*t*) is defined as the difference between the actual value *h*(*t*) and the predicted value, which is calculated as follows:

(2) $$\begin{array}{c} e\left(t\right)=h\left(t\right)-\dot{h}\left(t\right)=h\left(t\right)-\sum_{i=1}^{p}a_{i}h\left(t-i\right). \end{array}$$

LPC is obtained by minimizing the prediction error *e*(*t*), that is, the LP residual *r*(*t*) of the music signal. The analytical signal *r*_*a*_(*t*) can be obtained from *r*(*t*):

(3) $$\begin{array}{c} r_{a}\left(t\right)=r\left(t\right)+jr_{d}\left(t\right),\\ r_{d}\left(t\right)=IFT\left[R_{d}\left(\omega \right)\right], \end{array}$$

where *r*_*d*_(*t*) is the Hilbert transform of *r*(*t*); $R_{d}\left(\omega \right)=\left\{\begin{array}{cc} -jR\left(\omega \right), & 0\leq \omega <\pi \\ jR\left(\omega \right) & -\pi \leq \omega <0 \end{array}\right.$, *R*(*ω*) is the Fourier transform of *r*(*t*); *IFT* represents the inverse Fourier transform. Therefore, the analytical signal *r*_*a*_(*t*) can be expressed as

(4) $$\begin{array}{c} \varphi_{e}\left(t\right)=\left|r_{a}\left(t\right)\right|=\sqrt{r^{2}\left(t\right)+r_{d}^{2}\left(t\right)}. \end{array}$$

A lot of information about music emotion exists in the LP residual. Calculating the residual phase can help to extract the emotion-specific information in the music signal. The residual phase is the cosine of the analytical signal phase, which is calculated as follows:

(5) $$\begin{array}{c} \cos\left(\theta \left(t\right)\right)=\frac{r\left(t\right)}{\varphi_{e}\left(t\right)}. \end{array}$$

**Figure 2:**
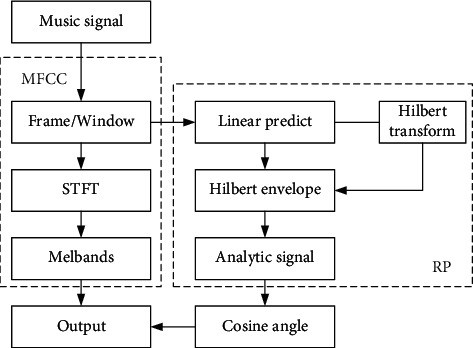
Feature extraction process.

The RP contains audio-specific information complementary to MFCC features. The recognition rate in the deep learning model shows that there is specific emotional information in the music signal, and RP can extract this specific information. The final output is obtained by weighted combination of MFCC features and RP features, which can improve the ability of the model to extract the emotional features contained in the music signal. The feature extraction process is shown in Figure 2.

### 2.3. CNN Emotion Classification

Audio is an important part of music. Most researchers analyze music emotion from the perspective of audio, generally extract time-domain and frequency-domain features from audio, and classify music emotion using traditional machine learning algorithms such as *K*-nearest neighbor, SVM, and Gaussian mixture model [22]. However, using the existing machine learning methods of frequency-domain and time-domain features is difficult to improve the performance of music emotion recognition. Because the manually extracted features usually belong to the low-level features of audio, these features are not directly related to the audio emotion state and contain less information. The high-level features of music are closer to emotion, and the amount of information is richer. However, the cost of manual feature extraction is higher, and some professional knowledge is needed. Some researchers apply the deep learning method to music emotion classification, and the experimental results are obviously better than the traditional machine learning method. Therefore, using the deep learning method can extract more emotion-related music features.

**Figure 3:**
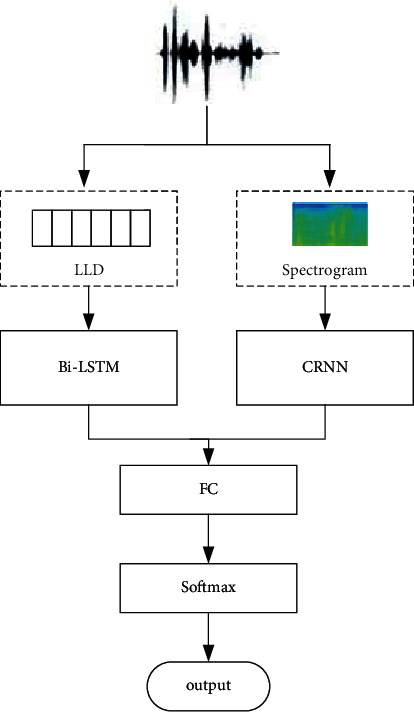
Overall framework of the music emotion classification model based on the improved CNN.

CRNN is the combination of the CNN and RNN, which is often used in sequence data modeling. Local perception ability of the CNN can fully extract the local features of data, and the RNN can effectively learn sequence information [23]. Music audio signal is a time series, and the previous frame and the next frame of audio are interrelated. Therefore, CRNN is applied to music emotion recognition, and a music emotion classification model based on the improved CNN is designed. This method combines the sound spectrum and the low-level features of audio as the input sequence. LLD is usually concentrated in the time domain or frequency domain. For the audio signal with associated changes in time-frequency characteristics, some information is often lost, while the sound spectrogram is a two-dimensional representation of the audio signal in frequency and has less loss in the amount of information. Therefore, LLD and sound spectrogram can realize information complementarity. The overall framework of the music emotion recognition model based on the improved CNN is shown in Figure 3.

As can be seen from Figure 3, the improved CNN structure is mainly divided into two parts, taking CRNN and Bi-LSTM as classification models, respectively. First, the spectrogram obtained from the audio signal is used as the input of the CRNN to extract the local and sequence features of audio. At the same time, the low-level audio features are extracted from the audio signal as the input of the Bi-LSTM part to further obtain the sequence information of the audio features. Finally, the audio features of the CRNN and Bi-LSTM are connected and input to the fully connected layer and then connected with the softmax classifier to output music emotion class labels.

By combining the CRNN model and Bi-LSTM model, a music emotion classification model based on the improved CNN is formed. The construction process of the model mainly includes three steps: (1) local feature and sequence feature extraction based on the CRNN; (2) sequence feature extraction based on Bi-LSTM; (3) music emotion classification based on softmax. Among them, the purpose of local feature and sequence feature extraction based on the CRNN is to extract the time-domain features, frequency-domain features, and sequence features of audio from the spectrogram for subsequent classification tasks. The main purpose of sequence feature extraction based on Bi-LSTM is to further extract the timing context information of audio from the low-level features of audio. At the same time, the low-level features of audio can complement the input information of the CRNN model [24].

#### 2.3.1. Local and Sequential Feature Extraction Based on the CRNN

The input of the CRNN part is spectrogram. Spectrogram is a two-dimensional representation of the audio signal in frequency, where the abscissa represents time and the ordinate represents frequency. The original audio signal is a one-dimensional array whose length is determined by the audio duration and sampling frequency. The audio signal is a nonstationary signal, but the spectral characteristics of the audio signal can be considered to be basically unchanged in a sufficiently short time. Therefore, during audio analysis, it is necessary to divide the audio into equal-length short segments, namely, audio frames, with a time length of 10 ms∼30 ms. After the audio signal is preprocessed by framing and windowing, fast Fourier transform (FFT) is performed for each frame, the time-domain signal is converted into the frequency-domain signal, and the transformed frequency-domain signal of each frame is stacked in time to obtain the sound spectrum.

**Figure 4:**
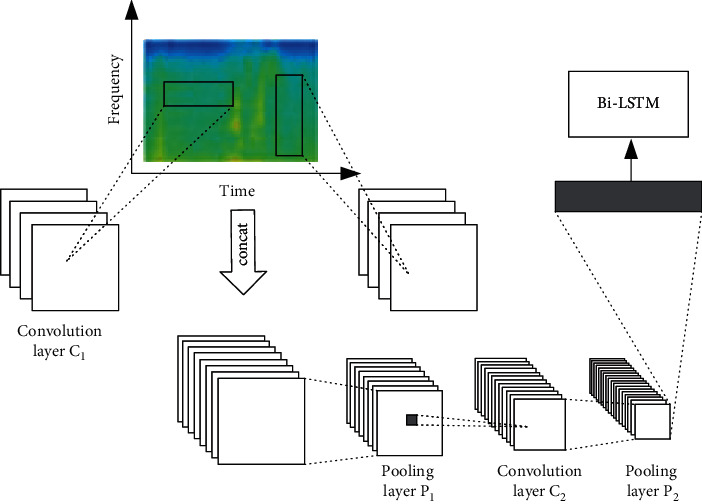
Network structure of the CRNN.

The basic structure of the CRNN is shown in Figure 4. First, the convolution layer *C*_1_ uses two different convolution kernels to extract the time-domain features and frequency-domain features of the spectrogram, respectively. The convolution kernel for extracting the time-domain features is convoluted in the time domain, and the convolution kernel for extracting the frequency-domain features is convoluted in the frequency domain. Then, all feature matrices of convolution layer *C*_1_ are concatenated and pooled, and convolution layer *C*_2_ further extracts local audio features. Finally, the feature matrices of the second pooling layer are taken out in turn and arranged into a vector as the input of Bi-LSTM to extract the timing information of audio features. In particular, both pooling layers *P*_1_ and *P*_2_ use max-pooling-over-time for downsampling.

**Figure 5:**
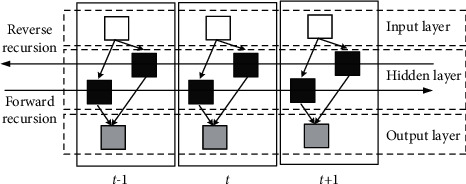
Network structure of Bi-LSTM.

The network structure of Bi-LSTM is shown in Figure 5. The core idea of Bi-LSTM is to construct two independent hidden layers. There is no connection between the two hidden layers, but the connection with the input layer and the output layer forms a set of LSTM. Each layer of Bi-LSTM independently learns the sequence dependence of input audio features, in which the forward sequence information of audio features is learned from the forward LSTM layer, and the backward sequence information of audio features is learned from the backward LSTM layer. The output layer concatenates the two hidden layer states into a vector, which is the timing feature of the input audio feature.

#### 2.3.2. Sequence Feature Extraction Based on Bi-LSTM

The input of the Bi-LSTM model is audio low-level features. The extracted audio low-level features include mel-frequency cepstral coefficient, fundamental frequency feature, formant feature, octave spectrum contrast, and chroma feature.

The two layers of Bi-LSTM independently learn the forward sequence information and backward sequence information of audio features and concatenate the two hidden states into a vector [25]. LSTM uses the gate mechanism to transfer information and update the memory state, which can learn long-distance dependent information and alleviate the gradient disappearance of the RNN. And Bi-LSTM can extract the temporal context features of the LLD in a short time.

### 2.4. Improved Softmax Classifier

#### 2.4.1. Softmax Classifier

In the scenario of using deep learning to realize multiclassification, softmax function is often used by many researchers. Softmax function can map the extracted feature to [0, 1], and the sum is guaranteed to be 1 through the normalization operation. The formula of softmax is

(6) $$\begin{array}{c} \phi_{y_{i}}\left(x_{i}\right)=\frac{e^{\varpi_{y_{i}}^{T}x_{i}+b_{y_{i}}}}{\sum_{i=1}^{n}e^{-i\omega t}}, \end{array}$$

where *ϖ*_*y*_*i*__ and *b*_*y*_*i*__ are, respectively, the weight and deviation of the last fully connected layer corresponding to the class *y*_*i*_ and *n* are the number of classes. For the objective function of multiclassification problems, the cross-entropy function is often selected, that is,

(7) $$\begin{array}{c} L_{\text{cross}}=-\sum_{k}y_{k}\log\;P\left(y=k\right). \end{array}$$

Intuitively, the standard softmax function enlarges the difference between input values with a natural base *e* and then normalizes the values into a probability distribution. In the classification problem, it is expected that the probability of the correct class identified by the model is close to 1, and the other probabilities are close to 0. If the linear normalization method is used, it is difficult to achieve this effect. The softmax function has significant advantages in the multiclassification problem by first expanding the difference and then normalizing. In the classification problem of the CNN, one-hot encoder is often used to process the predicted classes. At present, the general softmax function is to nonlinearly amplify each input *x* to exp(*x*) as follows:

(8) $$\begin{array}{c} \phi =\frac{e^{x_{i}}}{\sum_{j}e^{x_{j}}}. \end{array}$$

It can be seen from the above formula that the softmax function will separate the features of different classes, and there will be a certain distance between different classes, but the distance will remain unchanged when different classes approach to a certain degree. Therefore, the distance between the same class may be greater than that between different classes [26]. This problem is common in the field of face recognition. At the same time, for the multiclassification of music emotion, the training samples are limited. The ultimate goal is to classify the emotion of any unknown music. The actual test set is the concept of infinity. The above problems also exist, so the softmax classification function needs to be improved, while the softmax function ensures that emotion classes can be distinguished. More unknown data features should be considered to ensure that the feature vectors extracted during training are more compact among the same classes and more scattered among different classes.

#### 2.4.2. Introduce Variation of the Center Loss Function

In order to ensure that the classification model has the characteristics of intraclass convergence and interclass separation, some scholars have made corresponding improvements to softmax in recent years, including angular softmax and center softmax. The idea of angular softmax is to convert the separation characteristics between sample features into angle boundary learning. The specific formula is

(9) $$\begin{array}{c} L_{\Omega }=-\log\left(\delta_{g}\left(x\right)e^{L_{\theta }}\sum_{j}e^{L_{\theta }}\right), \end{array}$$

where *L*_*θ*_=‖*ϖ*_*y*_*i*__‖‖*x*_*i*_‖cos(*θ*_*y*_*i*__)+*b*_*y*_*i*__.

The weights of *L*_*θ*_ are normalized. Let ‖*ϖ*‖=1, and the offset is 0. The effectiveness of the function is verified by experimental analysis. However, these features still do not have good identification. With the increase of the amount of data, the improvement effect is limited.

The idea of center softmax is to minimize the intraclass spacing and control the feature center by introducing center loss. The specific formula is

(10) $$\begin{array}{c} L_{cs}=L_{s}+\tau L_{c}=-\sum_{i=1}^{m}\log\frac{e^{\varpi_{y_{i}}^{T}x_{i}+b_{y_{i}}}}{\sum_{j=1}^{n}e^{\varpi_{y_{j}}^{T}x_{j}+b_{y_{j}}}}+\frac{\tau }{2}\sum_{i=1}^{m}{\left\|x_{i}-Q_{y_{i}}\right\|}_{2}^{2}, \end{array}$$

where *Q*_*y*_*i*__ represents the feature center of the class, which will change with the change of features, *τ* represents the control factor of center loss, and *m* represents the size of mini-batch to update the feature center.

The music emotion classification belongs to the fine-grained classification, so it hopes that the final classification model can gather within classes and separate between classes. Center softmax only considers the centralization within the class, and there is room for improvement. Therefore, two improvements are considered: (1) the distance from the training sample to the class center is the shortest; (2) the sum of the distance between the training sample and its noncorresponding class center is the largest. The center loss function is improved by introducing the distance of noncorresponding classes. On the basis of controlling the center points of the same class, the distance between the center points of different classes is maximized as much as possible. The improved center loss function formula is

(11) $$\begin{array}{c} L_{\left(c/s\right)}=\frac{1}{2}\sum_{i=1}^{m}\frac{{\left\|x_{i}-Q_{y_{i}}\right\|}_{2}^{2}}{\left(\sum_{j=1,j\neq y_{i}}^{k}{\left\|x_{j}-Q_{y_{j}}\right\|}_{2}^{2}\right)+1}. \end{array}$$

The denominator plus 1 is to prevent the denominator from being 0. The final expression of the improved classification function center softmax is

(12) $$\begin{array}{c} L=L_{s}+\tau L_{\left(c/s\right)}=-\sum_{i=1}^{m}\log\frac{e^{\varpi_{y_{i}}^{T}x_{i}+b_{y_{i}}}}{\sum_{j=1}^{n}e^{\varpi_{y_{j}}^{T}x_{j}+b_{y_{j}}}}+\frac{\tau }{2}\sum_{i=1}^{m}\frac{{\left\|x_{i}-Q_{y_{i}}\right\|}_{2}^{2}}{\left(\sum_{j=1,j\neq y_{i}}^{k}{\left\|x_{j}-Q_{y_{j}}\right\|}_{2}^{2}\right)+1}. \end{array}$$

The improved loss function combines the center loss and the distance between classes, which can improve the distinguishability of features.

## 3. Experiments and Analysis

The experiments use the emotion music dataset to test and evaluate the performance of the proposed method in emotion recognition. The dataset consists of 2906 songs, which contain 4 emotion classes, including 639 anger songs, 753 happy songs, 750 relaxation songs, and 764 sad songs. For the convenience and uniformity of the experiment, only the first 30 s of each song is used, and those less than 30 s are filled with zeros. The dataset is randomly divided into three parts according to the ratio of 7 : 2 : 1, which are training, verification, and test sets, so as to maximize the fairness of the experiment.

### 3.1. Data Preprocessing

Mel spectrum is a common signal representation method in audio classification tasks. Compared with other advanced audio signal representation methods, mel spectrum retains the features of music signals more completely. At the same time, mel spectrum is more in line with human auditory characteristics. Therefore, mel spectrum is selected as the input data of music audio analysis.

**Figure 6:**
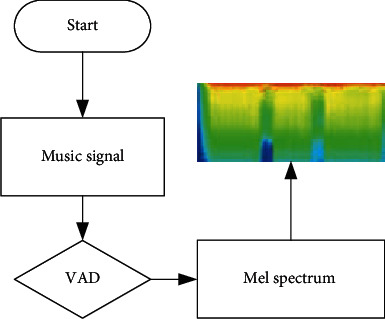
Preprocessing flow.

Voice activity detection (VAD) is to detect whether there is a mute frame in the music signal. These parts of the mute frame affect the recognition result. The flow of music audio signal representation and preprocessing is shown in Figure 6.

### 3.2. Audio Time Period Selection

Different researchers choose different lengths of audio clips. Therefore, experimental comparison will be carried out for different time periods, so as to select the most suitable audio time period and carry out subsequent experiments on this basis.

Experiments are carried out on the original CNN model and the improved CNN model. Audio clips of 3 s, 5 s, 10 s, and 16 s are selected for comparison based on the emotion dataset. The relationship between the time period and the number of samples is shown in Table 1.

As can be seen from Table 1, the length of the time period is inversely proportional to the number of data samples. The more the samples, the more comprehensive the coverage and the better the learning performance. Therefore, considering the relationship between the time period and the number of samples, the test is carried out on the first 30 s audio data of 2906 songs. The classification accuracy of the two classification models in different time periods is shown in Table 2.

As can be seen from Table 2, with the increase of time period length, the accuracy of model classification shows an upward trend, and better emotion classification performance can be obtained by using long-time audio data. In addition, the improved CNN model achieves higher emotional accuracy than the CNN model. Taking 10 s as an example, it is 6.56% higher. However, due to the limitation of data quantity of the emotion dataset, the use of audio data for a long period of time will greatly reduce the number of training samples, as shown in Table 1. By analyzing the experimental results in Tables 1 and 2, it can be seen that the learning performance of 10 s is the best. Considering the length of time period and the size of data samples, the time period of 10 s is selected as the division standard of the experimental audio signal, and the subsequent experiments are carried out on this basis.

### 3.3. Influence of Iteration Times on the Loss Value

**Figure 7:**
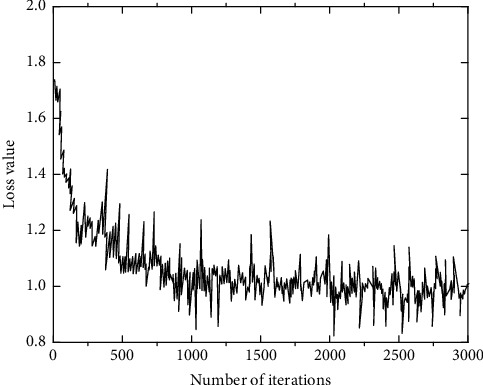
Influence of iteration times on the loss value.

The influence of different iteration times on the loss value is shown in Figure 7.

As can be seen from Figure 7, the greater the number of iterations, the more the number of weight parameter learning and adjustment, which can improve the accuracy of the model to a certain extent. With the increase of the number of iterations, the decline of the loss value gradually slows down and finally tends to be stable when the number of iterations exceeds 1500, which is about 0.98. Of course, too many iterations will also cause more computation. Through the analysis of results, the number of iterations is set to 2500 in the experiment.

### 3.4. Experimental Comparison of Different Convolutional Networks

The comparison between the lightweight CNN and classical CNN is mainly to illustrate the improvement of generalization ability caused by selecting and designing an appropriate lightweight CNN rather than using the classical network under the actual dataset. The two comparisons are not combined with knowledge distillation and transfer learning to ensure the independence of experimental comparison. The experimental results are shown in Table 3.

It can be seen from Table 3 that the classical CNN shows a high accuracy rate of 92.5% in the training set, but the accuracy rate decreases significantly by 11.57% in the test set. The lightweight CNN has achieved a high recognition accuracy of 85.54% on the test set, which can explain that there is overfitting under the CNN model and cannot have generalization ability on the whole dataset. The lightweight CNN can better solve this problem.

### 3.5. Emotion Classification Confusion Matrix

In order to further explore the classification of the music emotion state by the improved CNN model and measure the performance of the classification model, the emotion 4-class confusion matrices of the original CNN model and the improved CNN model are calculated, as shown in Table 4.

From the classification confusion matrix in Table 4, it can be seen that both the classical CNN model and the improved CNN model have the highest recognition rate for quiet and sad emotions. The classification accuracy of the improved CNN model for these two emotions has reached 87.93% and 92.54%, respectively. At the same time, the recognition accuracy of happiness and anger has reached an acceptable level, no less than 80%. In addition, the experiments show that the two emotions of happiness and anger are easy to be confused. Due to their similar expression in music, the improved CNN model can better distinguish the two emotions of happiness and anger compared with the classical CNN model, and the recognition accuracy of happiness is improved by 7.45%. Through the analysis of the classification confusion matrix, it can be seen that the stability of the improved CNN model is improved, and the improved CNN effectively improves the accuracy of music emotion classification.

### 3.6. Experimental Comparison of Different Classification Models

**Figure 8:**
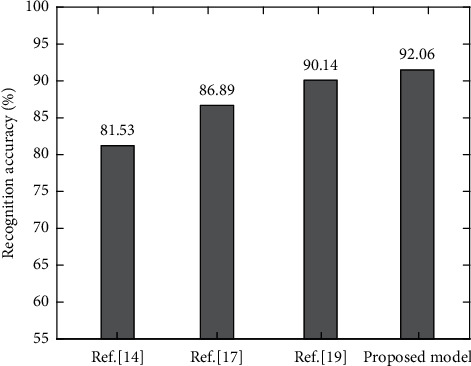
Recognition accuracy of different models.

In the comparison experiment, [14, 17, 19] are selected to compare and analyze with the proposed model on the emotion dataset. Take the average value of 10 test results as the final result, and the final comparison experiment results are shown in Figure 8.

As can be seen from Figure 8, compared with other models, the emotion recognition accuracy of the proposed model is the highest, reaching 92.06%. Because it uses CRNN and Bi-LSTM to extract music features, respectively, and integrates them into the improved softmax classification function to realize music emotion recognition, the recognition accuracy is well guaranteed. The improved loss function ensures the reduction of the distance within feature classes and increases the discrimination between different classes and enhances the adaptability of fine-grained image classification. [14] realizes music emotion recognition based on human physiological characteristics, in which support vector machines, decision trees, *K*-nearest neighbors, and multilayer perceptrons with different kernels are used to classify music, but because of relying too much on human characteristics, the method in [14] is not sensitive to emotion, the overall classification performance is poor, and the recognition accuracy is only 81.53%. [17] maps music acoustic features to corresponding arousal and titer emotion indicators through the linear regression model to realize emotion recognition, but audio feature extraction is not comprehensive enough, and the recognition accuracy is not ideal. [19] uses deep learning technology to identify emotions through musical instrument types and extracts features based on the RNN to identify emotions. The recognition accuracy is improved by 3.25% compared with [14], but the analysis ability of a single deep learning model is limited, and its recognition accuracy is reduced by 1.92% compared with the proposed model.

## 4. Conclusion

With the development of artificial intelligence and digital audio technology, music information retrieval has gradually become a research hotspot. Music emotion recognition has great research value for video music, but there is little research on it at present. Therefore, a music emotion recognition method using the convolutional neural network is proposed. The audio time-domain features, frequency-domain features, and sequence features extracted by the CRNN and the audio sequence context information of the Bi-LSTM extractor are fused and sent to the improved softmax classification function for analysis to identify four music emotion types. Aiming at the disadvantage of nonaggregation between classes in the softmax classifier, the proposed method introduces the center loss function to control the class center and increases the discrimination between classes. An improved softmax classification function is proposed to better distinguish similar emotions. The experimental results based on the emotion music dataset show the following:

1. The audio time period, the number of iterations, and the convolutional network structure will have a certain effect on the recognition model. When the time period of 10 s is selected, the number of iterations is set to 2500, and the lightweight network is adopted, the recognition performance of the model has been significantly improved.
2. Multimodal fusion can improve the accuracy of emotion recognition. The proposed model combines CRNN and Bi-LSTM to comprehensively extract music features. The recognition accuracy is as high as 92.06%, and the loss function value is only about 0.98, which can be well applied to music emotion recognition.

At present, deep learning method has gradually become the mainstream method of text emotion classification. Therefore, the deep learning method is considered to fully extract the emotional information of texts related with Chinese songs. In addition, the multimodal fusion method used in the proposed network is relatively simple and cannot fully extract the emotional correlation between audio and texts related with lyrics. How to achieve more effective multimodal fusion will be the focus of the next research.

## Figures and Tables

**Table 1:** Relationship between time period length and sample number.

Length of the time period (s)	3	5	10	16
Number of samples	23000	14000	7000	3000

**Table 2:** Classification accuracy in different time periods.

Length of the time period (s)	3	5	10	16
CNN model (%)	69.19	75.58	80.36	83.71
Improved CNN model (%)	76.83	81.06	86.92	88.25

**Table 3:** Experimental results using different convolutional networks.

Model	Recognition accuracy (%)	
	Training set	Test set
CNN	92.65	81.08
Lightweight CNN	89.71	85.54

**Table 4:** Confusion matrix of emotion classification.

Model	Emotion	Sad (%)	Happy (%)	Anger (%)	Quiet (%)
CNN	Sad	86.29	6.13	4.57	3.01
	Happy	7.05	76.43	9.28	7.24
	Anger	6.64	8.45	74.06	10.85
	Quiet	5.86	4.18	9.67	80.29

Improved CNN	Sad	92.54	3.06	2.28	2.12
	Happy	5.16	83.88	6.75	4.21
	Anger	5.19	5.26	80.07	9.48
	Quiet	2.78	2.95	6.34	87.93

## Data Availability

The data included in this paper are available without any restriction.
